# A New Division of Schizophrenia Revealed Expanded Bilateral Brain Structural Abnormalities of the Association Cortices

**DOI:** 10.3389/fpsyt.2017.00127

**Published:** 2017-07-20

**Authors:** István Szendi, Nikoletta Szabó, Nóra Domján, Zsigmond Tamás Kincses, András Palkó, László Vécsei, Mihály Racsmány

**Affiliations:** ^1^Department of Psychiatry, University of Szeged, Szeged, Hungary; ^2^Department of Neurology, University of Szeged, Szeged, Hungary; ^3^Department of Radiology, University of Szeged, Szeged, Hungary; ^4^Neuroscience Research Group, Hungarian Academy of Sciences, University of Szeged, Szeged, Hungary; ^5^Research Group on Frontostriatal Disorders, Hungarian Academy of Sciences, Department of Cognitive Science, Budapest University of Technology and Economics, Budapest, Hungary

**Keywords:** heterogeneity, subgroups, voxel-based morphometry, association cortex, heteromodal brain fields

## Abstract

The phenomenological and, consequently, pathophysiological heterogeneity of schizophrenia may be substantially decreased by determining etiologically valid subgroups. In a cross-sectional study, we analyzed the brain structural impairments of outpatients with schizophrenia using concurrent subgrouping methods, partly to enhance the extensity of exploration, and partly to estimate the validation of the divisions. High resolution T1-weighted MR images were obtained for 21 patients and 13 healthy controls. Localized gray matter volumetric deficits were defined with optimized voxel-based morphometry. Employing two concurrent methods (i.e., the widely known deficit-non-deficit division vs. the neurocognitive clusters we identified earlier) the patient group was iteratively divided into two subgroups, and their volumetric peculiarities were compared with one another and with healthy controls. Our division revealed more significant differences demonstrating bilateral brain structural deficits, which affected the association cortices, primarily the heteromodal fields and partly the unimodal fields. This is the first study that showed that abnormalities of the association cortices can be bihemispherial and expanded in schizophrenia, even in the cases of outpatients living integrated in society. Our result suggests that the extended association cortex abnormalities could constitute substantial and determining neurological substrates in the phenomenology and aetiopathogenesis of schizophrenia, at least in a subgroup of patients with more unfavorable neurocognitive characteristics.

## Introduction

From a research point of view, schizophrenia is widely accepted as a heterogeneous illness. Allowing for heterogeneity, the obviously non-overlapping clinical, pathophysiological, and etiological diversity can be substantially decreased by determining etiologically valid subgroups. Currently, only one division has a great impact, the division of deficit and non-deficit subgroups. The deficit syndrome has been defined as a putative subtype of schizophrenia by Carpenter et al. (1). According to this definition, the syndrome is characterized by primary, idiopathic, and enduring negative symptoms that are also marked and present during clinically stable periods. According to the authors, this distinction is not only a reliable and valid construct but also results in categorically distinct subgroups (2). As with other psychiatric diagnostic categories, the diagnosis of deficit syndrome shows minor (but relevant) instability. This observation was highlighted by the Amador et al. (3) follow-up study of diagnostic validity. This study found that when using a repeated diagnostic process, many years later, the initial diagnosis was actually changed to its opposite in 17% of the cases in the deficit group and in 12% of the cases in the non-deficit group. Furthermore, Peralta and Cuesta (4), who studied the distribution of temporary vs. permanent negative symptoms, and primary vs. secondary negative symptoms in a mixed group of psychotic syndromes (including those outside the diagnostic category of schizophrenia), found that deficit syndrome was not specific to schizophrenia. Depending on the diagnostic method used, persistent primary symptoms associated with the clinical diagnosis of schizophrenia were present in 14–37% of the cases, while their occurrence was 2–22% in other non-schizophrenic psychoses.

Aiming to detect valid schizophrenia subgroups, a robust cross-sectional study was performed in Szeged, Hungary. The study included performing a systematic neuropsychological battery, assessing clinical symptoms, neurological soft signs, and morphogenetic anomalies, and measuring event-related potentials of 50 outpatients with schizophrenia in their compensated states (5). An explorative fuzzy cluster analysis revealed two subgroups in this sample that could be distinguished from one another on symptomatologic, cognitive, and neurological levels. The analyses have demonstrated that cluster *Z* had more favorable characteristics, while cluster *S* had more unfavorable (more serious) characteristics. Importantly, these new clusters did not correspond to the deficit/non-deficit categories. In cluster *Z* (*N* = 27), 96.30% of the patients had non-deficit diagnoses, but in cluster *S* (*N* = 23) only 56.50% of the patients had deficit and 43.50% had non-deficit diagnoses. The differing patterns of detected cognitive dysfunctions and differences in the occurrence and the laterality of neurological developmental anomalies indicate that there may be hemispherical differences between patients belonging to distinct clusters. We characterized cluster *S* as a subgroup of patients with schizophrenia with lower education and IQ, which probably reflect a pronounced cognitive disorder (5). Specifically, these patients showed serious impairment in working memory and executive tasks, including assessments of visual working memory, attentional set shifting and inhibitory functions (5). Interestingly, the performance of cluster *Z* in these working memory and executive control tasks was not only significantly better than the performance of cluster *S*, but their performance was in the normal range of the population. In sum, patients belonging to cluster *S* showed a profound deficit in working memory and executive control functions, whereas the cognitive profile of cluster *Z* could be characterized with intact attentional and executive control functioning. Based on the analysis of the same patient population, we found less clear results with the deficit/non-deficit subgrouping method. The deficit and non-deficit subgroups did not differ significantly in tasks assessing working memory and inhibitory executive functions and showed only subtle difference in attentional set-shifting function, possibly due to the heterogeneity of the cognitive profile of patients in the non-deficit subgroup (5).

The structural MRI can reveal brain volumetric differences that might be related to the potential neural substrates of schizophrenia or represent the consequences of the disease. Although a plethora of studies have investigated brain volumes in schizophrenia, few publications have examined the differences between patients with deficit and non-deficit syndromes. Considering this division, one study (6) reported larger lateral ventricles on the right side in non-deficit syndrome compared to deficit syndrome, but it found no difference in terms of frontal lobe volume. However, the ventricles were found larger in deficit syndrome patients, according to some other studies (7, 8). The results regarding gray matter (GM) volume are also contradictory: non-deficit groups are reported to have less GM volume (9, 10), while other studies have found smaller GM volume in patients with deficit syndrome schizophrenia (11).

This study aimed to reveal the most brain structural anomalies in outpatients with schizophrenia, while concurrently using subgrouping methods for the patient group. The sample-population was composed of patients who had participated in the earlier cluster exploring study (5). In addition, the patients were in stable mental state at the time of this study, were cooperative, and were able to be matched to each other based on their age, gender, and clusters. The patient group was iteratively divided into two subgroups, and the brain volumetric peculiarities of the entire mixed group of patients and their subgroups were compared with the healthy controls and with one another in a cross-sectional study.

## Materials and Methods

### Subjects

Patients with schizophrenia (*N* = 21) were enrolled from the outpatient clinic of the Department of Psychiatry, University of Szeged. They were recruited from the previous cluster exploration study in which we explored cluster *S* and cluster *Z*; therefore, we knew the clusters for all the patients. Cluster *Z* had *N* = 10 patients, while cluster *S* had *N* = 11. Patients were diagnosed (DSM-IV-TR) (12) and clinically evaluated by an expert psychiatrist (István Szendi) using the Positive and Negative Syndrome Scale (PANSS) (13). The deficit and non-deficit diagnoses were made using The Schedule for the Deficit Syndrome (14); according to this measurement, there were *N* = 13 patients in the non-deficit group and *N* = 8 patients in the deficit syndrome group. All patients with deficit syndrome belonged to the cluster *S*. The patients were all in a stable condition and currently taking antipsychotic medication. All substances [eight types of second generation antipsychotics (SGA), one depot first generation antipsychotic, and one first plus second generation combination] were usually prescribed in moderate doses, according to the medication protocols; the mean dose in chlorpromazine equivalence was 282 ± 166 mg/die.

Control subjects were recruited from hospital staff and community volunteers. They were evaluated with a modified structured interview (Mini International Neuropsychiatric Interview) (15, 16). Control participants with a personal history of psychiatric disorder or a family history of psychotic and affective spectrum disorders, history of neurological illness, any medical illness known to affect brain structure, head injury with loss of consciousness for more than 30 min, clinically significant substance abuse within the last 6 months, or any medical illness that could significantly constrain neurocognitive functions were excluded. All participants were 18–50 years of age, with a minimum of 8 years of education (primary school), and able to give informed consent.

### Image Acquisition and Voxel-Based Morphometry (VBM)

Imaging was performed using a 1.5-T General Electric Signa Excite scanner at the Department of Radiology, University of Szeged, Hungary. High resolution T1-weighted images (3D IR-FSPGR, TR/TE/TI: 10.3/4.2/450 ms, flip angle: 15°, ASSET: 2, FOV: 25 cm × 25 cm, matrix: 256 × 256, slice thickness: 1 mm) were acquired.

We employed an “optimized” VBM-style protocol (17, 18) using FSL (19). Non-brain parts were removed from all structural images (20), and tissue-type segmentation was performed using FAST4 (21). The resulting gray-matter partial volume images were registered to a standard space (MNI152) using linear transformation (22), followed by a non-linear registration (23). The resulting images were averaged to create a study-specific template, to which the native GM images were then non-linearly re-registered. The registered partial volume images were then modulated (to correct for local expansion or contraction) by dividing by the Jacobian of the warp field. The modulated segmented images were smoothed with an isotropic Gaussian kernel, with a sigma of 3 mm. Finally, a voxel-wise general linear model was applied, using permutation-based non-parametric testing. The model encoded group membership. The following comparisons were carried out: patients vs. controls, deficit vs. non-deficit syndrome patients, *Z* vs. *S* cluster, and all subgroups vs. controls in separate analyses. Thresholding was performed using a novel method, the threshold-free cluster enhancing technique (24). Images were thresholded at *p* < 0.05 and corrected for multiple comparisons as implemented in FSL.

### Statistics on the Demographic and Clinical Variables

The statistical analysis of the demographic variables and the PANSS scores was performed using SPSS version 20 for Windows (released 2011, IBM Corp., Armonk, NY, USA). Years in education, IQ, age, duration of illness, and PANSS scores were compared by means of the non-parametric Mann–Whitney test. In the results section, Bonferroni-corrected *p* values were reported, although effect sizes were more pronounced as a result of the small sample size and high type two error rate. *R* values were calculated as indices of effect size.

## Results

### Demographics and Clinical Measures

Regarding the demographic variables, there was no significant difference between the healthy controls and the entire patient group or the different divisions of schizophrenia in terms of age, IQ (Wechsler Adult Intelligence Scale), and years of education. The duration of illness was similar in the schizophrenia groups. The PANSS scores indicated different clinical states at the time of the study. Cluster *Z* scored lower on the negative symptom subscale (Mann–Whitney *U* = 10, *Z* = 3.18, *p* = 0.01; *r* = 0.69) compared to cluster *S*. In the other classification, the non-deficit group scored lower on the negative (Mann–Whitney *U* = 6.5, *Z* = 3.31, *p* = 0.02; *r* = 0.72) and the general (Mann–Whitney *U* = 7.5, *Z* = 3.23, *p* = 0.02; *r* = 0.7) subscales, and they had lower total scores (Mann–Whitney *U* = 5, *Z* = 3.41, *p* = 0.01; *r* = 0.74) than the deficit syndrome patients. For exact data, see Table 1. We converted all antipsychotic doses to chlorpromazine equivalents using published equivalencies for conventional and atypical antipsychotics. No differences were found in the medication between the clusters or deficit and non-deficit groups.

**Table 1:** Demographic and clinical characteristics of the study groups.

	Healthy control		Subjects with schizophrenia																									
						Cluster					Deficit syndrome				
			Schizophrenia Group Total			*S*		*Z*			Deficit		Non-deficit		
							
	Median		Median		*r*	Median		Median		*r*	Median		Median		*r*
Education in years	12		11		0.25	10		12		0.56	9.5		12		0.47
IQ (Wechsler Adult Intelligence Scale)	108		99		0.38	92		103		0.61	89.5		103		0.65
Age	34		39		0.14	41		37		0.08	34		44		0.35
Duration of illness						15		13		0.15	13		15		0.13
PANSS-P						10		8.5		0.23	17.5		8		0.43
PANSS-N*^,^^#^						21		11		0.69	27.5		13		0.72
PANSS-G^#^						39		20.5		0.56	43.5		21		0.70
PANSS-T^#^						75		40.5		0.61	83.5		45		0.74

Gender	*n*	%	*n*	%		*n*	%	*n*	%		*n*	%	*n*	%	

Male	6	46.2	11	52.4		6	54.5	5	50		6	75	5	38.5	
Female	7	53.8	10	47.6		5	45.5	5	50		2	25	8	61.5	

*Significant difference (*p* < 0.05) between clusters.

*^#^Significant difference (*p* < 0.05) between the deficit and non-deficit groups*.

### GM Volume Alterations in Subjects with Schizophrenia

In this study, differences in brain structure between the healthy persons and the more favorable (the non-deficit and the cluster *Z*) subgroups of patients could not be detected (*p* > 0.2, corrected for multiple comparisons). In the same way, the differences between the more favorable and the more unfavorable (the deficit and the cluster *S*) subgroups of patients were not statistically remarkable (*p* > 0.2, corrected for multiple comparisons).

In addition to the whole group, the differences between the unfavorable subgroups of patients and the healthy controls reached the level of significance. In the cases of patients who belonged to either or both of the subgroups with more unfavorable natures (the deficit group and cluster *S*), there were verified notable brain structural differences compared to the healthy controls. Most of the relevant areas were revealed in the analysis between cluster *S* and the healthy controls.

#### Schizophrenia Group vs. Healthy Controls

**Figure 1:**
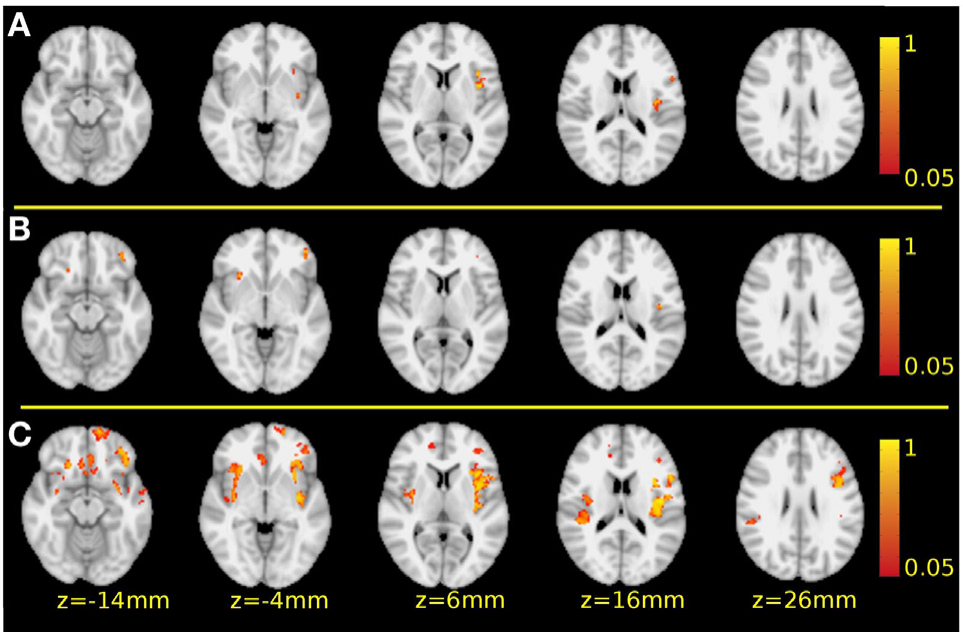
Significant gray matter deficits in schizophrenia patients. Row **(A)** the whole, mixed group of patients with schizophrenia compared to the healthy controls. Row **(B)** the deficit group compared to the healthy controls. Row **(C)** the cluster *S* compared to the healthy controls. Red-to-yellow volumetric clusters indicate *p* values between 0.05 and 1, corrected for multiple comparisons. In all three comparisons, the same slices are presented at *z*-coordinates indicated at the bottom of the figure.

The whole mixed group of patients with schizophrenia was measured to have lower GM volumes in the left insular cortex and left inferior frontal gyrus compared to the healthy controls (*p* < 0.036, corrected for multiple comparisons, Table 2; Figure 1—row A).

**Table 2:** Significant volumetric clusters of gray matter volume deficits in the group of patients with schizophrenia compared to the healthy controls.

Lobe	Region	Side	Size (voxels)	*p*_max_	*x* (mm)	*y* (mm)	*z* (mm)
Frontal	Inferior frontal gyrus	L	25	0.034	−54	12	14
Insula	Insular cortex	L	199	0.024	−40	12	8
	Insular cortex	L	77	0.026	−36	−16	20
	Insular cortex	L	23	0.036	−30	22	−8

*L, left*.

*The cluster sizes are indicated in voxels in standard space (voxel size: 2 mm^3^ isotropic). The maximal *p*-value is reported in the fifth column. Peak *p*-value coordinates are reported in the MNI152 standard space. There were three partly separable volumetric clusters detected in the area of the left insular cortex*.

#### Deficit-Syndrome vs. Healthy Controls

The deficit group showed lower cortical GM volumes in the bilateral perisylvian area, in the right orbitofrontal cortex and in the inferior and polar areas of the left frontal cortex (*p* < 0.05, corrected for multiple comparisons, Table 3; Figure 1—row B) compared to the normal controls.

**Table 3:** Significant volumetric clusters of gray matter volume deficits in the group of patients with deficit syndrome compared to the healthy controls.

Lobe	Region	Side	Size (voxels)	*p*_max_	*x* (mm)	*y* (mm)	*z* (mm)
Frontal	Frontal pole	L	114	0.029	−44	44	−8
	Inferior frontal sulcus	L	18	0.039	−36	42	8
	Orbitofrontal cortex	R	16	0.037	20	28	−16
	Orbitofrontal cortex	R	9	0.04	26	38	−8
Insula	Insular cortex	L	43	0.03	−40	−12	18
	Insular cortex	L	3	0.049	−38	−18	12
	Insular cortex	L	2	0.049	−34	12	12
	Insular cortex	R	37	0.032	28	22	−4
	Insular cortex	R	1	0.05	36	24	0

*L, left; R, right*.

*The cluster sizes are indicated in voxels in standard space (voxel size: 2 mm^3^ isotropic). The maximal *p*-value is reported in the fifth column. The peak *p*-value coordinates are reported in the MNI152 standard space. There were two partly separable volumetric clusters detected in the area of the right orbitofrontal cortex, as well as, three left volumetric clusters and two in the right insular cortex*.

#### *S*-Cluster vs. Healthy Controls

Voxel-based morphometry analysis indicated lower cortical GM volumes in the cluster *S* patients compared to the normal controls in the bilateral insula, bilateral orbitofrontal cortex, left superior temporal sulcus, right anterior cingulate gyrus, bilateral subcallosal region, and left inferior frontal sulcus and gyrus (the latter is the opercular part of the dorsal premotor cortex) (*p* < 0.047, corrected for multiple comparisons, Table 4; Figure 1—row C). Notably, the differences were more extensive than in the mixed group of patients or in the deficit subgroup.

**Table 4:** Significant volumetric clusters of gray matter volume deficits in the cluster *S* patients compared to the healthy controls.

Lobe	Region	Side	Size (voxels)	*p*_max_	*x* (mm)	*y* (mm)	*z* (mm)
Frontal	Frontal pole	L	302	0.011	−18	60	−8
	Inferior frontal sulcus	L	424	0.002	−42	6	26
	Inferior frontal gyrus	L	225	0.001	−54	12	12
	Orbitofrontal cortex	L	459	0.009	−34	34	−6
	Orbitofrontal cortex	L	352	0.014	−16	32	−20
	Orbitofrontal cortex	R	364	0.014	20	26	−14
	Orbitofrontal cortex	R	27	0.041	26	38	−10
Temporal	Superior temporal sulcus (planum temporale)	L	147	0.029	−44	0	−20
Insula	Insular cortex	L	2,774	0.003	−42	−12	16
	Insular cortex	R	2,714	0.014	34	−4	−2
Medial paralimbic cortices	Anterior cingulate cortex	R	63	0.042	14	48	8
	Subcallosal cortex	L + R	818	0.023	−2	24	−8

*L, left; R, right*.

*The cluster sizes are indicated in voxels in standard space (voxel size: 2 mm^3^ isotropic). The maximal *p*-value is reported in the fifth column. Peak *p*-value coordinates are reported in the MNI152 standard space. There were two partly separable volumetric clusters detected in the area of the orbitofrontal cortices on both sides. The affected areas of the perisylvian cortex on the right side seemed to be extending partly to the parietal lobe (onto the operculum and supramarginal gyrus), beside this the temporal deficits migrate lightly onto the parahippocampal areas*.

## Discussion

In the present cross-sectional study, we compared the brain volumetric characteristics of a mixed group of patients with schizophrenia to the structural relations of the healthy controls. Furthermore, using two concurrent methods (deficit and non-deficit vs. cluster *S* and *Z*), we divided the patient group into two subgroups, and structural imaging markers of these subgroups were compared with one another and with the healthy controls. Our results demonstrated the heterogeneity of the group of patients diagnosed with schizophrenia (according to the DSM-IV) who were treated in outpatient care, on the one hand, and the advantages of the subgrouping method in revealing brain structural anomalies on the other hand. Both subgrouping method uncovered more differences than the comparison between the whole patient group and the healthy control group.

**Figure 2:**
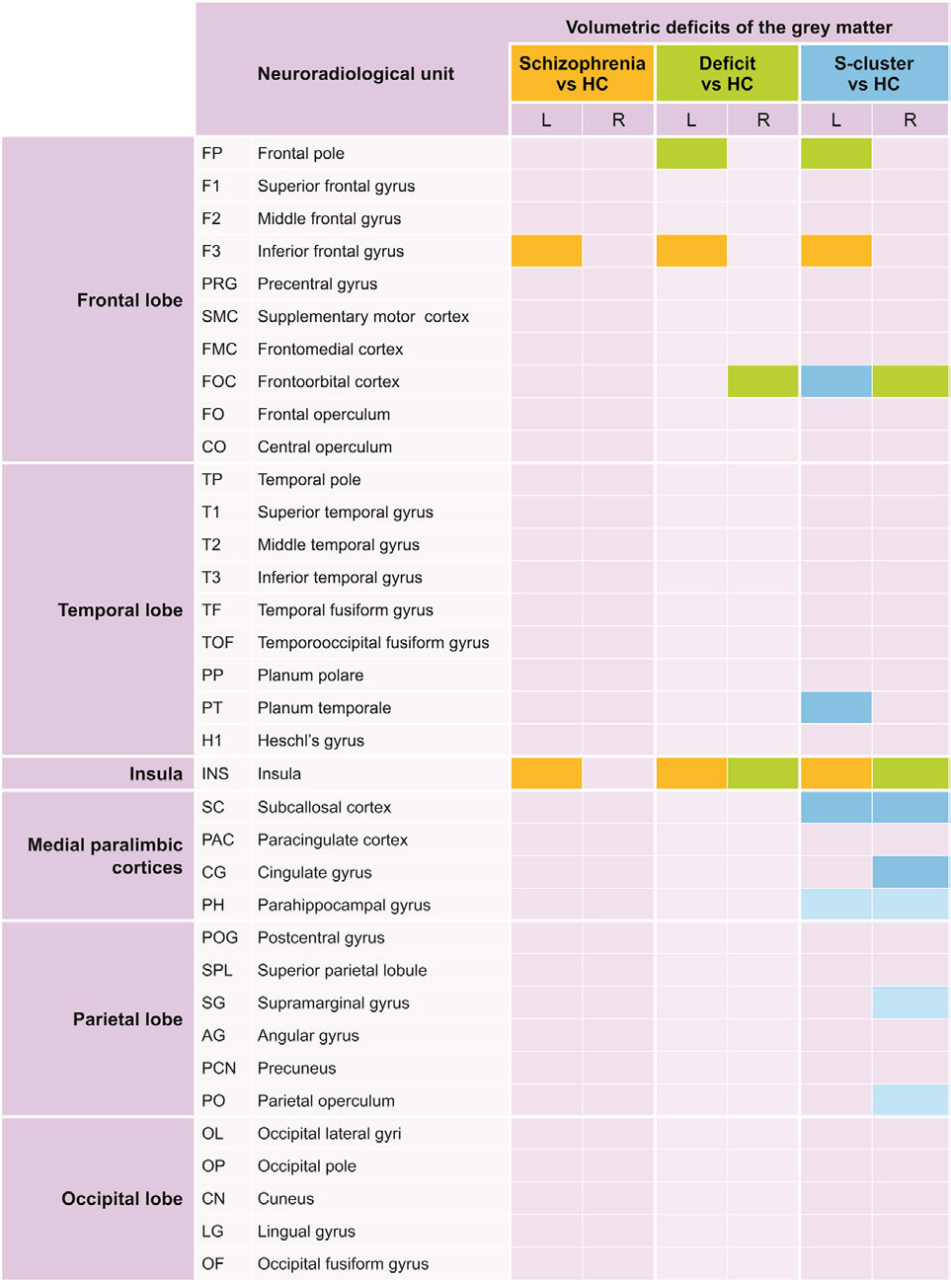
A graphical abstract of the volumetric impairments. The figure shows which cortical structures were affected in the different comparisons. The neuroradiological units are presented according to the work of Rademacher and colleagues (25). Alterations detected even on the level of the whole mixed group of patients (related to the healthy controls) are marked in yellow, green color marks alterations detected on the level of the deficit subgroup, and the anomalies detectable exclusively in the cluster *S* are marked in blue.

Based on our results, the brain volumetric impairments characterizing the patients most generally were the volumetric decrease of the inferior frontal gyrus and the insular cortex (both in the left hemisphere). These two volumetric deficits were observable in the whole mixed group of outpatients with schizophrenia; at the same time, these anomalies also appeared in the two unfavorable subgroups (in the deficit subgroup and in cluster *S*). In the subgroup of patients with deficit syndrome, in addition to the alterations in the whole group, volumetric decreases of the left frontopolar and right orbitofrontal cortices were detected and were accompanied by alterations of the insular cortices in both hemispheres. The latter anomalies were also pinpointed in the cluster *S*. Compared with healthy controls, the most expanded brain structural alterations were detected for cluster *S* patients. In addition to those anomalies that were also observed for the whole patient group and the deficit subgroup, there were volumetric deficits in the left orbitofrontal cortex, a decrease of GM volumes of the left planum temporale, right anterior cingulate cortex and of the bilateral subcallosal cortices. The perisylvian volumetric decrease on the right side expanded to the parietal lobe (onto the operculum and supramarginal gyrus), whereas the temporal impairment spread lightly onto the parahippocampal areas (Figure 2).

Based on the functional anatomy of these areas, the frontal pole and the orbitofrontal cortex belong to the frontal heteromodal association cortices. The inferior frontal gyrus and sulcus also partly belong to this area, and partly to the frontal motoric association cortex. The left planum temporale is a part of the auditory association cortex. The insula is a constituent of the paralimbic olfactocentric heteromodal association cortex. The anterior cingulum, subcallosal cortex, and parahippocampal gyrus belong equally to the paralimbic hippocampocentric association cortex. The supramarginal gyrus along with the parietal operculum is parts of the parietal heteromodal association cortex, and the latter participates in the constitution of the auditory and somatosensory association cortices as well. For details concerning the relationship of the affected regions and the association cortices mentioned in this paragraph, see the work of Rademacher et al. (25).

We can draw two conclusions from our results. First, although the involvement of association cortices could specifically differentiate the unfavorable subgroup of patients from the healthy controls, there could be no anomalies in the primary cortices. Second, the volumetric deficit in the association cortices (primarily heteromodal and to a lesser degree, unimodal) could be extended and could affect both hemispheres.

Although heteromodal cortex anomalies have often been theoretically associated with the aetiopathogenesis of the illness, this extent of involvement, to our knowledge, has never been detected. Through targeted examination of three heteromodal areas (prefrontal, inferior parietal, and superior temporal) in a mixed group of patients with schizophrenia and schizoaffective disorders, Buchanan’s team observed (26) volumetric decreases of the inferior prefrontal region and reversal of normal asymmetry in the inferior parietal cortex. In addition, few studies have empirically examined the supposed involvement of more than one heteromodal cortical association region as neuroanatomical substrates of the illness (27–29), moreover, with contradictory results. For instance, in one study, the volumes of the frontal and the superior temporal gyri were more strongly correlated in healthy persons (28), whereas another study found that the inferior parietal regions correlated with the prefrontal areas in patients but not in healthy persons (29). It is plausible that applying mixed, undifferentiated, heterogenic phenotypes could result in contradictory and ill-replicable results. Our results also suggest that targeted examination of association fields, with the help of neurocognitive subgrouping, could be promising in the explorative study of the aetiopathogenesis of schizophrenia.

The non-targeted, un-biased, data-driven VBM method allows examination of the whole cortical GM. VBM studies in schizophrenia have been frequently reported since the mid-1990s along with meta-analyses aimed at synthetizing and interpreting the data in the scientific literature of the field. One research group that builds analyses systematically focuses on brain structural alterations related to the genetic liability to psychosis and schizophrenia (30–33). Others deal narrowly with schizophrenia in comparisons between patients and healthy controls, or patients who experienced only one episode and those whose illnesses are chronic, or patients with schizophrenia and bipolar disorder (34–38). Besides, significant meta-analyses have pointed at the determining roles of gender (39) and laterality (40) in this research area.

The volumetric decreases of the left inferior frontal gyrus and insula, detected by our study in the whole, mixed group of patients with disease duration of 16 ± 7 years, are among the most frequently-reported VBM findings associated with the clinical expression of schizophrenia (37). Gray matter reductions of the left or bilateral insula were also observed in groups of patients with first-episode and chronic schizophrenia as well (34, 35). Moreover, these differences were revealed after the first psychotic episode in the broader, mixed samples of patients with psychotic disorders (32). Baseline volumetric decrease of the inferior frontal gyrus was also observed in high-risk subjects who later developed an established psychotic episode (30). Unlike in the case of clinical expression, no association with genetic diathesis was identified by a meta-analysis targeted at revealing VBM correlations of clinical manifestation and genetic liability (37). However, other meta-analyses could find correspondences volumetric decrease of the right insula relative to controls was detected in the group of first- or second-degree relatives of schizophrenic patients, qualified as a high-risk population by certain symptomatic signs or definite alteration of a genetic polymorphism (36). GM reduction of the left inferior frontal gyrus (associated with functional imaging hyperactivity) has also showed a connection to familial high risk for psychosis in a multimodal meta-analysis (33).

Bilateral volumetric reduction of the parahippocampal gyrus has also been consequently detected in the VBM meta-analyses and is suggested to be correlated to the genetic diathesis of schizophrenia (37). Moreover, reduction of volume of the PHG on the left side was associated with a genetic liability generally to psychosis (32), and the left-side decrease was also found by two meta-analyses comparing patients to controls (35, 40). Similarly, bilateral volumetric reductions of the anterior cingulum were found to correlate to genetic liability to schizophrenia (36) and more broadly to psychosis (32). Bilateral anterior cingulum reductions were also observable in studies comparing patients to controls (34, 35, 40). Additionally, an inverted laterality pattern was revealed in a targeted meta-analysis by Crow et al. (40) comparing patients with schizophrenia and bipolar disorder. GM reductions of the right anterior cingulum and left insula (and parahippocampal gyrus) were characteristic of schizophrenic samples, whereas the opposite pattern, volumetric decreases of the left anterior cingulum and right insula were observed in bipolar groups.

Interestingly, our neurocognitive subgrouping method has revealed some other GM volumetric alterations which have not emerged in earlier meta-analyses. All of them are considered parts of heteromodal association fields: the bilateral subcallosal and orbitofrontal cortices, the left frontal pole and planum temporale, and the right supramargynal gyrus and parietal operculum. Besides the above mentioned ROI studies [e.g., Ref. (26)], a VBM meta-analysis also suggested specific impairment of multimodal brain regions (37), assumingly with supervisory functions and with a determining role in the development of clinical manifestation, i.e., the impairment of the neural basis of the cognitive control system, which is one of the cognitive functions most-affected in this illness (41–43).

In the light of the VBM meta-analyses, our results can draw attention to one more relevant aspect: according to our results, in the case of chronic schizophrenia, the widespread cortical (34) and progressive cortico-striato-thalamic (36) GM decreases are not characteristic of all patients. They might play a pathogenetic role only in the subgroup of patients with more unfavorable neurocognitive capacities.

In summary, here we found that a cognitively unfavorable subgroup of patients with schizophrenia was characterized by an expanded, bilateral brain structural anomaly that affects the heteromodal and unimodal association cortices. This extent of distributed cortical volume deficit, which is specifically attached to the association areas, has not been detected in schizophrenia earlier. Note that patients belonging to this subgroup are living integrated into society, and are treated in outpatient care, that is, they do not belong to a particular ill sub-population.

Importantly, following the division of deficit/non-deficit syndrome, some patients with non-deficit syndrome also turned out to belong to this cognitively unfavorable subgroup (cluster *S*). Moreover, it is a surprising result that cluster *S*, which merged patients living with endurable primary negative symptoms (regarded as the most serious cases) with certain patients without deficit characteristics, revealed a larger magnitude of brain structural anomalies compared with the group of deficit syndrome. This result might show that in the case of patients with schizophrenia living integrated in the society the presence or absence of enduring, primary negative symptoms is not a solely etiologically relevant feature. It seems that the pattern of variables showing neurocognitive impairment can also be essential in revealing much more extended brain structural differences in the same pool of patients.

This result also indicates that in the case of patients without primary negative symptoms, pronounced cognitive and brain structural disorders are also crucial in revealing functional subgroups of this illness. Thus, in terms of neurocognitive deficits, a significant number of patients without deficit syndrome might be more closely related to patients with deficit syndrome than to other non-deficit patients with better neurocognitive functions. That is, according to our results, the subgroup of patients without deficit syndrome is fundamentally heterogeneous. Instead of assuming the primacy of the primary negative symptoms, the structural manifestation of the neurocognitive disorders appears to be more relevant.

This study has some limitations. Beyond the general difficulties of performing structural MRI investigations of patients with schizophrenia, where the observable volumetric deficit may be close to the limit of detection for MRI (44, 45), the number of subjects can also limit our results; a larger patient cohort might improve the reliability of the results. The sample-population was composed of patients who had participated in the earlier cluster-exploring study (5) for placement into clusters. In addition, the patients had to be in a stable mental state at the time of this study, had to be cooperative, and had to be able to be matched to each other based on their age, gender, and clusters. This was the barrier which determined that we could not enroll more patients. Another potential limitation is that external and/or repeated validation of the existence of our subdivision of patients to clusters *S* and *Z* using robust systematic neurocognitive mapping is unavailable for the time being. Furthermore, in addition to the genetic heterogeneity, the treatment heterogeneity of patient populations (46) must be considered. Moreover, for the time being, we cannot establish a clear understanding of the influences that are exerted on brain structural alterations by the chronic use of antipsychotics in schizophrenia. Some meta-analyses suggest that we should take these influences into account [e.g., Ref. (47, 48)]; however, it is not clear enough what the extent of these impacts is, what kind of regional or agent- or duration-dependent peculiarities they have, and how all of these can be manifested on the bases of the individual dynamics of the progression of structural changes in the brain. The heterogeneity of the disorder makes our clairvoyance in this area difficult as well. Because this illness is also heterogeneous regarding the progression of structural changes in the brain (49, 50), the influence of persistent therapy cannot be evaluated clearly on the basis of studies executed on samples of mixed patient groups. It is not surprising that a systematic review could not find a linear relationship between the degree of antipsychotic exposure and progressive structural changes in the brain (51). Furthermore, specific pharmacological agents and types of agents can exert different influences in this area. It seems that applying first-generation antipsychotics (FGA) is associated with more unfavorable consequences: according to the results of a meta-analysis based on longitudinal studies, patients treated with at least one FGA higher-mean daily antipsychotic dose correlated with more progressive GM loss, while in patients treated only with SGA less progressive GM loss was detected in the case of higher daily doses (52). A meta-analysis detected brain regional differences related to treatment: in four areas a relative volumetric decrease appeared, while in three areas a volumetric increase was observable (53). Among these, only the volumetric decrease of the left inferior frontal gyrus occurred in our study as well; there were no other overlaps with the other regions. Considering the number of aspects needed to evaluate that in this study, in the subgrouping divisions, the CPZ equivalent doses of the antipsychotic therapy applied at the time of examinations did not correlate with the cross-sectional brain structural differences.

Clarifying the phenotype of schizophrenia is a relevant and urgent step in revealing the etiology of the illness. Defining subgroups through a theoretically robust and complex set of neurocognitive variables could help to detect more alterations than examining mixed groups or symptomatic subgroups of patients determined only by clinical phenomena and diagnoses. Based on the results of this brain structural study, we can suggest several hypotheses. The neurocognitive subgrouping method devised by us earlier (cluster *S* and *Z*) seems to be a more relevant dividing method than the deficit-non-deficit separation. The subgroup of patients without deficit syndrome seems to be fundamentally not homogeneous. In the more unfavorable neurocognitive subgroup of patients (cluster *S*), an expanded bilateral brain structural impairment could be detected through volumetric analysis that primarily affects the heteromodal association cortices. This is the first study that showed that abnormalities of association cortices can be bihemispherial and expanded in schizophrenia, even in the cases of outpatients living integrated in society. The extensive abnormalities of the association cortices could constitute a peculiar, substantial and determining neurological substrate in the phenomenology and aetiopathogenesis of schizophrenia, at least concerning a subgroup of patients with more unfavorable neurocognitive characteristics. Targeted examination of association fields in neurocognitive subdivision seems to be promising.

## Ethics Statement

This study was carried out as a part of a research entitled “Brain MRI study, and neurocognitive assessments in psychosis spectrum disorders, Human Investigation Review Board, University of Szeged, Albert Szent-Györgyi Medical Pharmaceutical Centre” (No 2655) in accordance with its recommendations. All subjects gave written informed consent in accordance with the Declaration of Helsinki.

## Author Contributions

The study was conceptualized by all of its authors. Author IS designed the study and wrote the first draft of the manuscript. NS and ZK managed the MR investigations, analyzed the imaging data, and drafted the work’s second manuscript. IS managed the clinical assessments and interpreted the results. ND managed the recruitment all patients and healthy controls with IS. She also managed the descriptive analysis and assisted with writing the first draft. MR assisted with interpreting the results and writing the final draft of the manuscript. AP and LV supervised the planning and execution of the study and the manuscript in terms of radiology and neurology, respectively. The authors came to an agreement to be accountable for all aspects of the work in ensuring that questions related to the accuracy or integrity of any part of the work are appropriately investigated and resolved. All authors approved the final manuscript to be published.

## Conflict of Interest Statement

The authors declare that the research was conducted in the absence of any commercial or financial relationships that could be construed as a potential conflict of interest.
